# Application of Drug and Exercise Intervention in Postoperative Rehabilitation: A New Evaluation of Health Coordination Effect

**DOI:** 10.3389/fsurg.2022.909425

**Published:** 2022-04-21

**Authors:** Weide Shao, Qian Wang, Tian Liao, Qiaoyin Tan

**Affiliations:** ^1^ College of Physical Education and Health Sciences, Zhejiang Normal University, Jinhua, China; ^2^ College of Humanities and Foreign Languages, Hunan Agricultural University, Changsha, China; ^3^ College of Teacher Education, Zhejiang Normal University, Jinhua, China

**Keywords:** drug intervention, exercise intervention, collaborative intervention, postoperative rehabilitation, integration of sports and medicine

## Introduction

Postoperative rehabilitation is an important process of physical function recovery after surgery. Surgery is an effective means to treat sport injuries, cardiovascular diseases, cancer and other physical diseases. However, some major operations are still accompanied by postoperative complications, such as pain, infection and physical dysfunction. Therefore, postoperative rehabilitation has become an important means to restore physical function and health after surgery. Rehabilitation includes a series of activities, such as pharmacology, exercise, nutrition, diet and social psychology (1). A large number of studies showed that drug and exercise intervention play an important role in postoperative rehabilitation.

**Figure 1:**
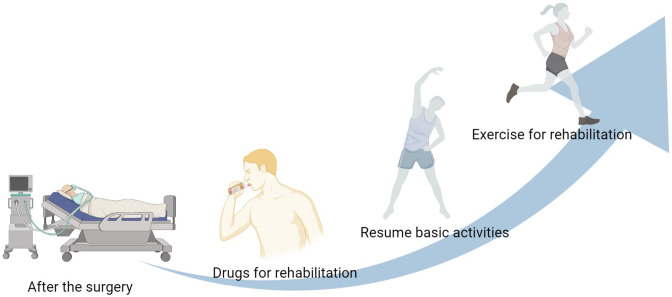
Postoperative rehabilitation process.

Postoperative pain is a normal phenomenon after surgery, which requires drug intervention to relieve the pain. Some drugs have anti-inflammatory, analgesic and nutritional effects, such as dexamethasone, lidocaine, vitamin B12 and normal saline (2). It can relieve postoperative edema, pain and infection. There are also some analgesic drugs with few adverse reactions, such as non-steroidal anti-inflammatory drugs and opioid partial agonists, which also play an important role in relieving postoperative pain. Exercise therapy refers to rehabilitation therapy based on functional exercise. It can promote the energy metabolism of musculoskeletal, prevent joint spasm and slow down osteoporosis. At the same time, it can also improve the muscle strength and exercise endurance of the body. After operation, the patient's physical function is greatly reduced, and complications may occur. Rehabilitation is the continuation of surgery, which can restore the patient's physical function. As shown in Figure 1, patients receive treatment with some rehabilitation drugs. With the relief of body pain, they can do some basic physical activities.

A large number of studies have shown that drug and exercise intervention play an indispensable role in postoperative rehabilitation. In order to provide new ideas for the development of postoperative rehabilitation, this paper systematically studies the application of drug intervention, exercise intervention and their collaborative intervention in postoperative rehabilitation.

## Postoperative Rehabilitation Methods

### Drug Intervention

Postoperative drug intervention is mainly used for anti-inflammation and analgesia. Traditionally, the main methods of postoperative analgesia are oral analgesia by intravenous injection of opioids and patient-controlled analgesia (PCA). Although opioid analgesics have certain effects, they usually cause side effects, for example, nausea, vomiting, itching, dizziness, insanity, bladder and intestinal dysfunction, etc (3). Some experience has been summarized in clinic for better pain management and accelerate patient rehabilitation. Some experience has been summarized in order to do a good job in pain management and accelerate the rehabilitation of patients clinically. For example, multi-mode labor analgesia is adopted, i.e., different labor analgesia techniques are combined and different analgesics with different action mechanisms are applied, so as to exert the additive or synergistic effect of analgesia.

Periarticular multimodal drug injection (PMDI), which included ropivacaine, epinephrine, and ketoprofen. It can effectively relieve postoperative pain and reduce the consumption of analgesic, and promote postoperative rehabilitation (4). Multimodal analgesia can be combined with pre-operative analgesic administration, for example, it can be composed of paracetamol, opioids and Cox-2 inhibitors. This approach is to reduce pain by preventing peripheral and central hypersensitivity reactions. Peripheral nerve block (PNB) is also used to control postoperative pain (3). Based on the fact that multiple drugs are mixed for nerve block during the operation, some researchers have proposed the method of impregnating drugs with gelatin sponge to increase the concentration of liquid around local nerve roots to treat reactive pain due to nerve root edema in the early stage after operation. Drug mixtures used include ropivacaine, dexamethasone, and vitamin B12 (2). This method also has certain limitations, such as repeated pain after surgery and drug dependence.

Drug intervention is no longer limited to one drug or method for postoperative rehabilitation. The combination of multiple drugs can improve the efficacy, but there are also certain side effects.

### Exercise Intervention

Exercise has the effect of a “multi-effect drug”. Moderate and regular physical activity can reduce the occurrence of cardiovascular disease. Improvements in cardiovascular health and functional capacity can accelerate postoperative recovery and reduce mortality (5). Exercise is an important complement to all surgery. The types of exercise intervention are: aerobic exercise, resistance exercise, aerobic combined resistance exercise, home exercise and multi-mode exercise.

Resistance training is one of main components of postoperative rehabilitation and musculoskeletal system injuries. Exercise interventions for early postoperative patients after lung transplantation, mainly including power cycling, walking and resistance exercise, showed significant improvements in walking distance, lower limb muscle strength and quality of life (6, 7). In a one-year randomized controlled trial, a combination of high-intensity repetitive training and endurance training (cycling) significantly improved knee joint function after degenerative meniscectomy over other exercise programs that emphasized seperate elements (8). Muscle volume and strength will decrease significantly in older patients after surgery. Although high-intensity resistance training may pose a risk for musculoskeletal injury (9), chronic resistance (CR) exercise and chronic aerobic (CA) exercise are conducive to the postoperative functional recovery and reduction of complications in elderly patients (10). Blood flow restriction (BFR) therapy, as a postoperative exercise intervention, has attracted wide attention in recent years. BFR therapy can stimulate muscle hypertrophy with benefits for cardiovascular health and postoperative pain (11).

Scientific and reasonable exercise intervention has the characteristics of high safety. It has a certain degree of relief and stability on postoperative pain in patients. Exercise intervention has increasingly become an effective way of postoperative rehabilitation.

### Collaborative Interventions

Collaborative intervention refers to postoperative rehabilitation under the joint action of drugs and exercise. The goal of postoperative rehabilitation is to restore range of motion, intensity, endurance and body function while avoiding complications. Single technologies and drugs cannot eliminate postoperative morbidity and mortality. The combination of medication and exercise therapy may have a better effect on postoperative rehabilitation.

The use of postoperative analgesic drugs was controlled in two groups of experimental patients. Patients took non-steroidal anti-inflammatory drugs twice a day for the first 7 days. For the remaining 14 days, the dose of celecoxib was reduced to 100 mg. The group that started ROM on the first postoperative day showed significantly less postoperative pain than the group that started ROM on the seventh day (12).

While drug intervention in the early postoperative period played a role in analgesia and reducing inflammation, exercise is needed in the later postoperative period to better facilitate body recovery. Exercise earlier after surgery may also relieve pain.

## Conclusion

(1) The effects of drug intervention and exercise intervention on postoperative rehabilitation have been confirmed by a large number of studies. Postoperative patients suffer from physical pain and decreased body function, and they must rely on drugs and exercise to recover health. (2) Drugs are used for postoperative rehabilitation, with the main purposes of anti-inflammation and analgesia. A mixture of multiple drugs was often used for intraoperative nerve block to relieve postoperative pain. Exercise intervention is mainly used to restore the body function, and it is also possible to avoid complications caused by postoperative long-term lying. (3) Both of them have some limitations and side effects while promoting postoperative rehabilitation. For example, long-time postoperative administration of analgesics may result in drug dependence, and inappropriate rehabilitation exercise may hinder wound healing or even lead to secondary injury. Drug and exercise synergy may work better in postoperative rehabilitation.

## Discussion

Drugs and exercise play an extremely important role in postoperative rehabilitation. The form and effect of a single drug or exercise need to be improved. Synergistic intervention of drugs and exercise is more conducive to the postoperative rehabilitation of patients, but at the same time, it puts forward higher requirements for the selection of drug dosage and exercise. There are few related studies. In the future, we should pay more attention to the role of exercise in the prevention of disease, and develop personalized and targeted disease prevention exercise prescription.
